# From centralization to cooperation: The development and reform process of sports venues in China from 1949 to 2022

**DOI:** 10.3389/fspor.2022.1077211

**Published:** 2023-01-06

**Authors:** Xuemo Fang, Yuanxin Chen, Jingyu Li

**Affiliations:** ^1^ College of Physical Education and Health Sciences, Zhejiang Normal University, Jinhua, China; ^2^ School of Physical Education, Central China Normal University, Wuhan, China; ^3^ National Sports Industry Research Base, Central China Normal University, Wuhan, China; ^4^ Youth League Committee, Wuhan Sports University, Wuhan, China

**Keywords:** government-enterprise relationship, operation and management, reform, sports in China, sports venues

## Abstract

The management of sports venues has undergone a series of reforms since the People's Republic of China's establishment in 1949. The reform of management right is especially significant. It reflects the government–enterprise relationship and logic of government action. Utilizing the perspective of the government–enterprise relationship, this study systematically reviews the reform model for sports venues to incorporate Chinese characteristics. The aim of this study is to understand the relationship between the People's Republic of China's government and the market through the reforms implemented for the operation and management of sports venues. According to the study, the development and reform of sports venues in China has experienced government centralization, devolution, decentralization, and cooperation. The reform of sports venues' operation and management follows a “market-oriented government-led” model which reflects the government's logic. It is concluded that a “market-oriented government-led” model is essential for the promotion of comprehensive reforms for sports venues with Chinese characteristics.

## 1. Introduction

In China, sports venues are symbols of not only sports development but also of the development of competitive and mass sports. Since the establishment of the People's Republic of China (PRC), local governments have stressed the development of sports and invested heavily in the construction of sports venues. However, due to their large size and high cost, the operation and maintenance of sports venues have always been recognized as a problem, worldwide. Most Chinese sports venues are state-owned assets that are mainly operated and managed by public institutions. With the continuing reform of the Chinese economic system, the management rights of sports venues have been gradually liberalized, and their reform and classification as public institutions has persistently advanced. The operation of an increasing number of venues has followed a market-oriented approach through, for example, entrustment management, service outsourcing, and joint venture, which is more flexible and advantageous in terms of aspects such as institutional mechanism, talent introduction, and market operation. Nevertheless, the government has played an essential role in reforming the operation and management of sports venues. In the absence of market players, the government has assumed the functions of the market and actively facilitated the emergence of market entities to create a good business environment.

## 2. Manuscript formatting

### 2.1. Purpose of research

At different historical periods, the relationship between the government and the market has embodied different characteristics. This is particularly evident in the development and evolution of sports venues, which has reflected China's unique development patterns and laws. Therefore, assessing the process of the development and reform of sports venues highlights the characteristics of the relationship between the Chinese government and enterprises. The historical analysis adopted in this study will enliven the understanding of the reform of sports venues and facilitate the formulation of better strategies to cope with new development opportunities and challenges. Thus, this study analyses China's experience of the reform of sports venues' operations and management, focusing on the relationship between the government and the market, it systematically compares the development and reform of venues over the past 70 years since the founding of the PRC, and summarizes the reform path of venues operation and management with Chinese characteristics, which provides a useful reference for sports venue management in other developing countries.

### 2.2. Method

Utilizing the government–enterprise relationship perspective, this study systematically reviews the reform model for sports venues to incorporate Chinese characteristics. The historical analysis adopted in this study will enliven the understanding of the reform of sports venues from 1949 to 2022 in China. Data for analysis included public policies, laws, document compilations, government reports, internal meeting materials, and other public as well as unpublished data.

### 2.3. Results

Based on the characteristics of the government's power over the market, the process of development and reform is divided into the following four stages: centralization, devolution, decentralization, and authorization. Table 1 shows the four stages of the development and reform process of sports venues in China.

**Table 1:** Four stages of the development and reform process of sports venues in China.

Stages	Government-Enterprise Relations	Reform model and characteristics	Achievements	Issues
Centralization Period (1949–1977)	Government Replacing the Market	1) government owned all the sports venues and directly managed and protected them; 2) sports venues had a strong military and political orientation	State-owned assets of venues are effectively protected.	Single supply body of venues, closed management system, and inflexible mechanism
Devolution Period (1978–1991)	Government Allowed Market Access	1) Diversification of venue supply entities; 2) Contracting Responsibility System for Sports Venues; 3) Differential Budget Management	The economic benefits of venue operations have been improved, increasing revenue and saving expenses.	There is a lack of self-managed, self-financing market players, the balance between public welfare and operation rights is unclear, and there is a loss of state-owned assets.
Decentralization Period (1992–2012)	Demarcation of the Boundaries between the Government and Enterprises	1) Non-public economic entities participate in the supply of venues; 2) Adopt a modern enterprise system for venue management; 3) Dual system for public institutions and enterprises; 4) Diversified business development strategy for venues	Market dynamics have been enhanced, and market-oriented reforms have been standardized.	The supporting system is not perfect and the resistance to reform is high. Excessive commercialization and lack of public welfare in the process of venue operation.
Cooperation Period (2013-present)	Government proactive strengthen of market entities	1) Entrusted management-based management rights reform; 2) Shareholding reform of state-owned enterprises; 3) The quality and expansion of venue services and consumer upgrades	Introduce market competition mechanism to achieve optimal allocation of resources; stimulate social forces to participate in investment and operation; stimulate and guide the development of sports consumption market.	The venue management reform model is not clear enough, and the division of labor and cooperation model between the government and the market is not clear. In the reform process, there is a lack of professional market players, an imperfect bidding mechanism, and a lack of strong supervision.

#### 2.3.1. Centralization period (1949–1977): government replacing the market

At the initial stages of the establishment of the PRC, China experienced a period of comprehensive socialist transformation and peaceful construction, during which it established a centralized political system and planned economic system. At this stage, the government attached great importance to the development of sports; strengthening people's health was an important political task undertaken by the party and society. The promulgation and implementation of the “Preliminary Labor and Provisional Regulations on the Sports System of Defending the Country” has extensively promoted the large-scale development of sports training and competitions in various regions and stimulated the enthusiasm towards investing in the construction of sports venues.

With systematic planning and administration, sports venues were built quite efficiently. In fact, 261 new sports venues were constructed before 1978, including a brief period of significant growth between 1956 and 1960 wherein 17 new sports venues were constructed annually (1). Local sports administration departments were the main stakeholders in the construction of these sports venues. In 1960, to reduce this dependence on the (local) government, the Physical Culture and Sports Commission (PCSC) proposed the diversification and socialization of the supply of sports venues. Following this initiative, other departments, such as the education department, began actively building sports venues. As of 1978, the education and other departments had constructed 112 and 27 sports venues, respectively (2). Although the supply pool was diversified, it remained restricted to the state-owned and collective economy, where financial allocations by the government were the main source of investment.

During this period, the government owned all the sports venues and directly managed and protected them. For example, the Shanghai Municipal Government took over and protected 31 sports venues which were either publicly owned, foreign-owned, or privately owned venues in the former concession area during the old Shanghai period and carried out maintenance and renovation using state funding (3). These sports venues continued to operate under the management of the PCSC in the new period. The management and operation of sports venues was subject to the strict implementation of the budgetary management requirements for public institutions to ensure “uniform revenue, unified expenditure and unified management” (4). For the venues that generated an income, the financial figures of turning in and subsidies were determined, while the portion that was not covered by this revenue was included in the national budget. The strict budgeting was established to facilitate business development and site maintenance after review rather than to increase the number of staff and their wages.

Due to the national atmosphere at the initial stages of the establishment of the PRC, sports venues had a strong military and political orientation (5). They were mainly used for military events, such as the People's Liberation Army Sports Games and the People's Liberation Army Shooting and Review Conference. They also hosted large-scale political activities and sports activities that included drills, reviews, and performances, including the National Workers' Sports Congress, National Games, Ethnic Minority Performance Congress, and other sports activities. Moreover, the national sports system experienced a brief period of military takeover between 1968 and 1973 (3). The operation and different forms of utilizing the sports venues, at all department and unit levels, were also mainly open to use by employees and students within the system of their respective units, and it was difficult to realize universally open services outside those units.

Due to the characteristics of the “all-involved” national organizations in China, sports venues relied on powerful administrative artillery for their construction or transformation in the case of limited financial resources. Therefore, the government utilized public financing to fund the operating expenses of venues and ensured the protection of state-owned assets by establishing nationalized and unified management representing a government-led venue management model. Additionally, in the context of war threats and political crisis during the initial days of the PRC, sports venues became important symbols of the achievements of national politics and defense.

As the centralized government was the only entity managing the investment in and building of venues, investor and builder attributes were relatively uniform across the venues of this period. Although they were efficient for short-term construction, they were not sustainable because the market did not facilitate survival. Essentially, the management system was closed, and there was no room for enterprises to survive. Due to the uninformed management system, inflexible mechanism, and highly centralized financial management system—which restricted the autonomy of venues' operation and management—it was difficult for the egalitarian distribution system to stimulate the staff's enthusiasm.

It can be said that the development of sports venues in this period reflected the national will of the centralized power. However, according to the history of the sports industry, the construction and operation costs of sports venues were substantial. Therefore, the revolutionary impulse to catch up has replaced the rational construction system. Neither the market nor society had been effectively developed, or when they were developed, they failed to perform their roles; this affected how sports progressed in China (6).

#### 2.3.2. Devolution period (1978–1991): government allowed market access

During the early stages of reform and opening up of the economy, the Central Committee of the Communist Party of China (CCCPC) recognized the serious defects of “excessive centralization of power” (7) in the Chinese economic management system. Focusing on the requirements of economic construction, the government proposed that “Local and industrial and agricultural enterprises should be given more autonomy in management and administration under the guidance of the unified plan of the state” (7). Therefore, the central government decentralized the administrative power to the local units and implemented a fiscal responsibility system to improve the economic conditions for the establishment of the local government's autonomy and enthusiasm. Meanwhile, the government delegated power to production units that depended on administrative agencies, expanded their operational autonomy, implemented reforms related to the contractual management responsibility system in state-owned enterprises, and approved the application for the internal corporate management of public institutions in some sectors. These measures laid the foundation for the reform of the management system of sports venues.

In 1984, on the implementation of the “Notice of CCCPC on the Further Development of Sports”, the PCSC observed the following: “we should let go of mobilizing the whole society to run sports” (8). The source of funding for the construction of venues was not limited to financial appropriations. Since the time of the reform and liberalization, the non-public sector of the economy developed rapidly. Private and foreign-funded enterprises started occasionally investing in sports venues and providing sponsorships and donations. According to the data of the 3rd National General Survey of Sports Venues, of the 523,130 newly built sports venues nationwide, the PCSC accounted for 2.7%, while investment by the education and other departments accounted for 97.3%. Foreign joint ventures, fundraising, and Chinese people who lived overseas, self-employed investment, and construction accounted for about 0.005%. During this period, the construction of the sports venues was part of the local economic and social development plans and urban construction plans. Local governments mobilized social forces to operate sports through various channels by absorbing foreign and private capital, especially in the eastern coastal areas such as Guangdong and Fujian that take advantage of the regional competition and recognize the importance of the private economy for investment and the construction of venues (9).

In 1988, the PCSC issued the “Opinions on the Trial Implementation of Contracting Responsibility System for Sports Venues” (10), encouraging, for example, complete or partial contracts or leases of sports venues while mandating the completion of tasks such as training, competitions, and mass activities established by higher-level departments. This helped in fully realizing the potential of the venue to embody various functions. Several venues opened their doors and diversified operations to effectively improve economic efficiency and accumulate funds for the development of sports. Therefore, funding for sports venues has transitioned from being state-sponsored to generating operating income. For example, the Capital Gymnasium was operated using a 300,000-yuan financial subsidy before 1984. After the implementation of the contract responsibility system, the income increased by eight or nine times by 1990 (11). “Supporting the main business by side-line” model was successful in mobilizing employees' enthusiasm. In 1991, the annual average self-sufficiency rate of China's public stadium funding income reached 65% (12)..

The PCSC and the financial department had implemented the balance budget management system with “full management, fixed income and expenditure, balance subsidy (or handed in), guaranteed use and balance retention” (13) for sports venues. In this system, the tasks, establishment, operational targets, and funding subsidies are determined according to the scale and number of venues. Meanwhile, the use of excess income and savings expenditures was liberalized. In addition to the maintenance and equipment purchases, a proportional commission from the profit was allowed to be used for collective welfare and rewards for employees. For example, during the reform of the Shanghai sports venues, the labor income of employees was linked to the operating results, and the assessment indicators and distribution methods were rationalized. This significantly improved the employees' enthusiasm and efficiency of management.

The government's decision to devolve powers and profits revitalized the nearly suffocated national economy under the planned economic system and established an institutional foundation for the reform of the operation and management of sports venues. With the diversification of the suppliers of sports venues, balanced budget management was essential to increase income and reduce expenditure, and a contract responsibility system was thus employed to improve economic efficiency. However, social forces that participated in the construction of sports venues did not have a substantial impact during this period. The primary supplier of sports venues remained within the governmental system. Therefore, devolution was limited to the administration. It did not consider enterprise autonomy; consequently, enterprise reform was still in the exploratory stage. Although the direct intervention of the government in the market was reduced, construction of the corresponding system was missing. It was difficult to implement effective supervision for short-term behaviors in the process of venue contracting and operation because prominent problems occurred after decentralization. In pursuit of economic benefits and profits, some contractors undertook non-sports related projects such as cultural performances, exhibitions, and circuses, changing the intended use of the venue and occupying public welfare sports service time. Some operators displayed behaviors such as making arbitrary charges and increasing prices. As a result, the original structure of some venues was destroyed, resulting in sizable property losses. Social benefits were difficult to guarantee, and the masses continued to complain, causing several negative effects. In short, the government's decentralization has an obvious incentive effect on venue reform. Therefore, market regulation merely played an auxiliary role in this period. The reform did not embody independent management and self-financing market entities, and the relationship between public welfare and the economic benefits of the venues remained unclear.

#### 2.3.3. Decentralization period (1992–2012): demarcation of the boundaries between the government and enterprises

In 1992, the 14th National Congress of the Communist Party of China (NCCPC) established the reform goal of establishing the socialist market economy system and proposed that the market mechanism should play a fundamental role in the allocation of resources. “Separation of government from enterprise”, “separation of government affairs”, and “separation of management and operations” (14) became important reform directives for state-owned enterprises and public institutions. The Tax-Share Reform, established in 1994, was completely contrary to the local fiscal contract system. It went beyond the traditional reform ideas that delegated power and transferred profits. This reform marked the beginning of the exploration of reforming from within the system and the emergence of the management advantages of the modern corporate system.

In addition to the government, schools, and other institutions, domestic and foreign private enterprises began participating in the construction of sports venues. In the context of the central government's reduction of the subsidies for the construction of sports venues in various regions, local governments boldly and innovatively raised funds. For example, during the preparations for the 8th National Games in Shanghai, the local government used multiple strategies such as land replacement, differential land rent, rolling development, multi-function development, and the utilization of foreign capital to raise funds for the construction of venues (15). Bird-Nest, the Beijing Olympic National Stadium, was the first large-scale stadium in China that was constructed under the public–private partnership (PPP) model (16). The project used the legal person bidding method to establish the CITIC Consortium as the main body involved with investment and financing, construction, operation, maintenance, and handover in the form of a franchise. As the sponsor and owner of the project, the government provided support in the form of, for example, land, funds, and taxes. Although the consortium gave up subsequent franchising shortly after the investment and construction (after the Beijing Olympics), the model ushered in a new era of government-led, market-operated sports venues in China.

In 1993, the PCSC's policies named “Opinions on Cultivating Sports Market and Accelerating the Process of Sports Industrialization” (17) and “Opinions on Deepening Sports Reform” (18, 19) established the requirement of “gradual industrialization of sports institutions” and “shifting from welfare type, public welfare type, and government-affiliated type to business type”. If the conditions permitted, the sports institutions could become economic entities and implement corporate management. Based on the policies, some sports venues began exploring the corporate internalization of public institutions. The management system of the Hongkou Sports Venues in Shanghai was reformed in 1993 by drawing on the experience of modern enterprise management, establishing the organization, clarifying the functions and powers of each department, and forming a management system in line with the characteristics of modern enterprises and sports venues, which standardized the internal management. This became the basis of implementing the job contract system and internal wage system, as well as establishing venture capital contract workers, which mobilized the staff's enthusiasm. At the same time, the enterprise initially understood the separation between ownership and management rights, realized independent operation and self-financing, and then formulated the development model of “Unified management, decentralized management, collective benefit, more work and more gain” (20), signified by cooperative contracting and collective management. Ultimately, the stadium achieved the goal of “Not eating the imperial grain to pay the public grain” and became a symbol of the national stadium reform (20).

To improve the efficiency of operation, some venues established state-owned venue operation enterprises to retain the original institutions, that is, “two brands and one team” of institutions and enterprises. For example, the Shanghai Hongkou Sports Stadium established the Shanghai Hongkou Football Stadium Lu Xun Park Joint Development Group on the basis of the original institution in 1999 and later renamed it the Shanghai Changyuan Group, responsible for the corporate operation of the stadium. In 2005, Wuxi City established the Municipal Stadiums and Sports Training Management Centre (MSSTMC) as the owner of state-owned assets. These were authorized by the municipal government to perform the investor's responsibilities on behalf of the government and manage the assets, personnel, and business of the entire market. After the reform, the original local sports bureau was no longer responsible for the specific business of the venues but for macro-management, such as policy planning and operational guidance. Additionally, a completely state-owned company was established and entrusted by the MSSTMC to conduct “market-oriented operations, corporate management, social services” for stadiums in Wuxi City (21). Wuxi's innovative “separation of management and operation” model enabled the Sports Bureau and the Sports Management Centre to perform their respective responsibilities. This effectively separated administrative management and professional development and realized the organic unity between public welfare and profitability.

Some venues were classified as institutions that required restructuring. For example, in 2006, Shenzhen restructured four government-sponsored institutions responsible for the management of municipal-owned sports venues (21). After the restructuring, the assets completed the enterprise industrial and commercial registration, merged into the state-owned asset supervision system of municipal enterprises, and carried out enterprise entity operation. However, due to the lack of supporting policies on personnel and assets, it was difficult to transfer business affairs to enterprises, which often led to the neglect of the public welfare of venues. Therefore, the institutional reforms initiated in 2006 were not effectively implemented.

Under the guidance of the full utilization of venue resources, diversification become an important operation strategy in this period. Mainly through internal development, strategic alliance, entrusted management, the diversified commercial development and operation of a venue implied its use for fitness and leisure, cultural and sports activities, and ancillary space (22). This was not only the multi-operation of content and service in the traditional sense but also reflected the overall strategic planning of the organizational structure, business model, and business philosophy. For example, the Jiangsu Wutaishan Sports Centre implemented comprehensive reforms in 1995. In the process of diversifying operations, this sports center has continuously adjusted and formed a relatively complete organizational structure system as well as relatively mature management models and operating concepts. The sports center's operating system includes more than a dozen subsidiaries, such as nationwide fitness and large-scale theatrical performances, extensive development of advertising, product scale, sports construction and other businesses, and the implementation of related diversified business strategy. The subsidiaries support each other, their costs and risks are shared, and customers, marketing channels, and brand benefits are shared as well. This creates synergy and strengthens the overall market competitiveness of the center (23). However, the diversified management model of most venues was not completely mature, and the problem of excessive commercial development was pervasive. While the business scope of the venue is wide, its relevance is weak, and its cost and risk cannot be shared by the subsidiaries because of the absence of effective early-stage demonstrations, matching operating mechanisms, and organizational structures. Instead, it increases the operational burden and makes it difficult to achieve economies of scale.

The “Hongkou Model”, “Wutaishan Model”, and “Wuxi Model” were significant explorations of local stadium reform which reflected the gradual improvement of the understanding of China and its market mechanisms as well as the promotion of the practice of local stadiums' operation and management. At the same time, the promulgation of the “Law of the PRC on Physical Culture and Sports” and the “Public Cultural and Sports Facilities Regulations” (24) provided a legal framework for promoting the development of stadiums, regulating their reform and development, and maintaining public welfare. If the stage of “devolution” represented the supply of sports venues and the management mechanism along with the government’s “concession of profits” (an external incentive) that belonged to the market, then the stage of “decentralization” represented the re-establishment of the relationship between the government and the market. This stage included the self-management of stadiums and the production of intrinsic motivation. The decentralization and diversification of the management of stadiums increased the market's vitality and generated income. However, the excessive emphasis on economic benefits neglected the social benefits and deviated from the original intention of making the stadium open to the public (25). Economic indicators for internal contracting were rather arbitrary and lacked scientific basis and objective standards. In addition, due to the imperfect supporting system, the institutional reform of public institutions demonstrated greater resistance. Finally, the “one-size-fits-all” type of reform was likely to cause the excessive commercialization of a stadium, with no consideration of public welfare and the persistence of residual problems such as personnel placement.

#### 2.3.4. Cooperation period (2013-present): government proactive strengthen of market entities

With the gradual improvement of the market economy system, the reform associated with this era entered a critical period. For the first time, China's 12th five-year plan proposed that “more attention should be paid to the top-level design and overall planning of reforms” (26) that reflected the “regulations first” characteristic of reforms during this period. The comprehensive revision of reforms centered on strengthening system construction was a re-examination of the relationship between the government and the market. The government transitioned from the “government-enterprise unity”, wherein the government substituted the market and directly managed venues, to “government–enterprise separation”, wherein the government delegated power and profit and encouraged market growth, and finally to a “government–enterprise cooperation” with clear functional boundaries and emphasis on government services and supervision. Following the decentralization reforms, the government began examining itself and ultimately limited its power.

The enterprise reform of sports venues was an important channel for the reform of sports venues' system and innovation of mechanisms during this period (27). In 2013, the General Administration of Sport of China (GASC) and eight other ministries and commissions jointly issued the “Opinions on Strengthening the Reform and Innovation of the Operation and Management of Large-Scale Sports Venues and Improving the Level of Public Services” (28), and in 2014, the State Council issued the document named “Several Opinions on Accelerating the Development of the Sports Industry and Promoting Sports Consumption” (29). Both policy documents proposed the introduction and use of the modern enterprise system to stimulate sports venues' vigor.

The marketization of management rights was at the core of the enterprise-oriented reform of venues. By handing over the management rights to the enterprises, the modern enterprise system can be used to improve the operation efficiency of venues. In 2016, the National Development and Reform Commission (NDRC) and 24 other ministries and commissions issued the “Action Plan on Promoting Consumption-Driven Transformation and Upgrading”. This plan proposed that the venues of administrative agencies and public institutions should introduce social capital and modern corporatization operating mechanisms to understand that venues are state-owned and the management rights belong to the company. Subsequently, this reform model was successively included in China's “13th Five-Year Plan for Sports Development” and “13th Five-Year Plan for Sports Industry Development”, and Jiangsu, Zhejiang, and Chongqing were selected as pilot areas to explore and promote reforms. In 2018, the nationwide pilot project entitled “Reform of functions and reform mechanisms”—which represented reform related to the operating rights—was launched, emphasizing the reform of the functional applicability of venues to create favorable conditions for the reform mechanism.

The reform of management rights was realized in various ways. In 2019, the “Opinions of the General Office of the State Council on Promoting National Fitness and Sports Consumption to Promote the High-Quality Development of the Sports Industry” emphasized that the government's investment in new stadiums should be operated by third-party enterprises without establishing a separate institution for management. For the existing venues, it was mainly realized through multiple modes such as the dual-track of business and enterprise, state-owned with state-owned, and state-owned with privately owned. However, in the reform practice, an increasing number of venues choose the entrusting management mode to understand the separation of the two rights and gradually replace the modes of independent operation, leasing, contracting, and so on. The contractual relationship between the owner and the operator of the venue constitutes the core of entrusted management. Because the contract stipulates the rights and responsibilities of both parties, a more mature, stable, and cooperative relationship was established between the government and the market. In addition, the government—the client of the venue management right—implemented effective incentives and constraints on the venue operators through contract realization clauses. This led to the venue operators considering the interests of their clients and realizing the effective operation of the venues. This also embodied the government's functional transformation from direct operation and management to oversight and incentives for the market through contracts.

The joint-stock reform with multiple property rights effectively stimulated the vitality and competitiveness of venues. According to the “Guiding Opinions on the Definition and Classification of State-Owned Enterprises”, most of the sports venues in China are state-owned enterprises under the public welfare category and generally operate as completely state-owned enterprises. Those which met the conditions were eligible to promote the diversification of investment entities. It was also possible to encourage non-state-owned enterprises to participate in operations through purchasing services, franchising, and entrusting agents. This provided an institutional basis for the joint-stock reform of sports venues. During this period, according to the degree of joint-stock reform of venues, the following two reform practices existed.

First, the joint venture model and joint-stock cooperation were used to introduce strategic investors to compensate for the lack of funds, technology, talent, and other resources and achieve mixed management rights. For example, the Shanghai Mercedes-Benz Cultural Centre was developed and constructed by a joint venture company comprising state-owned enterprises which authorized AEG-OPG Culture and Sports (Shanghai) Co., Ltd. to operate and manage the project. This operating company was a Sino-foreign joint venture aimed at providing insight on the professional management of venues through the rich operating experience of foreign-funded enterprises.

The second reform practice was the PPP model. New venues widely utilized the build–operate–transfer (BOT) and operate–transfer (OT) models and employed social forces to participate in the investment, construction, and operation of the venues. The government determined the social capital of venue investment through competitive negotiation. This was followed by the operation, management, and market cultivation of the venue by the joint venture company that was established by the state-owned enterprises. During the operation period, a combination of user fees, government purchase services, and feasibility gap subsidies were adopted to ascertain the return of social capital on investment and income. For existing venues, the renovate–operate–transfer (ROT), OT, and other models were widely used, and social forces were introduced to rebuild and operate venues.

These two reform practices have brought a new mode of cooperation between the public sector and private enterprises, which not only provides an important way for social forces to participate in sports stadium investment and increase the enthusiasm of private capital participation, but also realizes the participation of multiple subjects, reduces government risks and eases government financial pressure. This is a reflection of the modernization of the government's governance capacity and governance level. It can be believed that the reform is not only a victory for private enterprises, but also a victory for the cooperative relationship between the government and the market.

In addition, equity incentives and employee shareholding, open asset restructuring, government guidance funds, asset securitization, and debentures were also effective ways to reform the state-owned enterprises' shareholding system. Currently, these are being explored and used in stadiums. In 2015, the Wutaishan Sports Centre also proposed ideas pertaining to “employee stock ownership” and stating that “conditions are ripe to start the IPO process” for the corporate reform plan. Jiangsu, Zhejiang, and other places changed the original government investment method and use of fiscal funds by establishing sports industry guidance funds, wherein funds were used as leverage to attract more social capital to participate in the investment of state-owned venues and realize the diversification of equity.

Sports consumption was listed as one of the six major consumption growth areas in the 2015 government work report. In 2016, 24 ministries and commissions, including the NDRC, jointly issued a notice that suggested creating an action plan to promote consumption-led transformation and upgradation. Sports fitness consumption was listed as one of the “top ten consumption expansion actions” and a concrete request was put forth for the full activation of the venue resources. The “Action Plan for Further Promoting Sports Consumption (2019–2020)”, which was promulgated in early 2019, established the concept of “expanding the space of sports consumption” (30), encouraging the integrated development of fitness, leisure, business services, culture, tourism, and other industries to promote sports consumption. Many venues began innovating business models to create an urban activity center (31) and expand sports, culture, leisure, business, and other urban functions to achieve an organic combination. Jiangsu province formulated the guidelines for accelerating the construction of sports service complexes and identified such complexes for three consecutive years. It fostered several well-developed sports consumption clusters with distinctive industrial characteristics that were leading and demonstrating. This included sports-center-type and national-fitness-center-type complex projects cultivated around venues which inspired innovation in the sports venues industry. In 2020, the GASC selected several typical cases of the sports service complex, out of which 13 were transformed from venues, including large-scale sports venues that embodied competition performance as the leading role, and small and medium-sized sports venues that took national fitness as the leading role. This provided a very good sample for optimizing the service function of sports venues and promoting the marketization management level.

This period marked an innovative era for the operation and management model. First, the reform of the management right disbanded the traditional management model of venues under administrative thinking, creating conditions where the market mechanism could play a decisive role. An increasing number of venues are introducing market competition through public bidding for the right to operate and achieve the optimal allocation of resources. Second, the cooperation between the government and enterprises widely exists in the investment, construction, and operation of venues. Many newly built sports venues adopted the PPP model to realize the transformation from government-arranged management to market-sharing and introduce non-state-owned capital as well as a flexible mechanism. Innovative concepts of enterprises regarding the venues were successful in stimulating the enthusiasm of social forces to participate in investment and operation. At this period, most venues have achieved both social and economic benefits through reforms, but according to Chen et al. (2022) (32), we believe that there are still some venues where economic benefits have increased while social benefits have decreased.

### 2.4. Discussion

China has always viewed the relationship between the government and the market dialectically; however, it did experience a period under planned economy wherein the government completely replaced the market. This was followed by the government taking efforts such as bold decentralization, clarification of the boundaries between the government and enterprises, and the gradual withdrawal from the market so as to establish a new relationship with the market. Based on the empirical evidence from China's economic development, it can be concluded that the government's role is not smaller or better than the market. In fact, it played a significant and far-reaching role. In-depth analysis of China's reform and development path reveals that the government's initiatives have always been centered on the development goals of marketisation and other market-oriented characteristics. The Chinese government has been proactive and active and had played a significant role in the process of market development. At the same time, the government's actions were not limited by the administrative logic. Instead, it has respected market law and used market-oriented administrative measures to achieve market development.

China's reform of the operation and management of sports venues signifies a “government-led” perspective, but this does not mean that the market mechanisms were ignored. On the contrary, the market mechanism was transformed from “unable to play an effective role” to playing an “auxiliary role” (33) in the period of the planned economy and then a “basic role” (34) and a “decisive role” (35). It was observed that the reform of the operation and management of sports venues revolved around the changing relationship between the government and the market. Initially, the government proposed a “social force to run sports” and encouraged public sports venues to improve their management by actively opening them up to the outside world. In the 1990s, the government recognized the development requirements of sports marketization and industrialization, which was followed by the innovation of the management system, mechanism, and reform of the operation and management of venues in the 2000s. The reform of the operation and management of sports venues represented a “market-oriented government-led” characteristic which is a culmination of historic and theoretical knowledge.

In contrast to “government-led marketization”, which emphasizes the role of government in the marketization process, “market-oriented government-led” highlights the fact that the government-led process focuses on the long-term marketization development goals, and the administrative measures taken also have obvious market-oriented thinking and characteristics. In the critical period of transition to a modern market economy, the government needs to deepen institutional reforms across the board, including adjusting economic development goals, government focus, and the scope of its authority, and the government still needs to be active and proactive across the board. The government's role is to protect macroeconomic stability, strengthen public services, maintain a fair competitive market environment, and to respect market laws, cooperate with market behavior, utilize market mechanisms, and maintain market order. In the period of China's economic transition, government-led is a necessary and reasonable transitional arrangement, especially in the early stage of development, China cannot naturally form a market mechanism like European and American countries, and needs to intervene through the government, directly allocate resources and gradually cultivate the market. Specifically, “government-led marketization” is mainly manifested in three characteristics, which is clearly different from a “market-oriented government-led” approach.

First, the law of the market was respected and transformed from “administrative logic” to “market logic”. In “government-led marketization”, the market plays a subordinate role and is dependent on the government. The “administrative logic” dominates the process. However, the “market-oriented government-led” perspective embodies a deeper understanding of market laws. The acceptance and application of the system reflects the utilization of both the national system and the market mechanism (36). Respect for the law of the market is manifested in the following three aspects: improving the relationship between supply and demand, emphasizing value creation, and protecting market competition.

The second characteristic was to create an institutional environment to transition from “policy-driven development” to “systematic development”. “Government-led marketisation” involves the one-way decentralization reform of the market which highlights the unilateral role of the government. However, the top-down and inside-out paradigm of decentralization is problematic. Because most of the reform results come from the intervention of the administrative department, they are an easy tool with which to strengthen intervention, making it difficult for reform to deepen. In the “market-oriented government-led” perspective, the government gradually enhances market enthusiasm through decentralization and forms a property right operation mode compatible with the market economy through the decentralization of the government. While the government's control (old powers) was gradually decentralized, transferred, or obliterated, a new relationship of function and power was established between the government and market under the “limitation of powers”. The transformation of the paradigm of government decentralization is manifested through the institutionalization of government power, rationality of policy implementation, and tendency of government–enterprise interaction.

The third characteristic was to cultivate the market entities and transition from “empowered enterprises” to “energized enterprises”. “Government-led marketization” stimulates market vitality through the gradual decentralization of power. A “market-oriented government-led” perspective does not imply the blatant transfer of power in the hands of the market to “decrease one and the other”. Rather, under the reform of “double power”, a new type of symbiotic relationship is constructed between the government and the market, meaning that both the “active government” and “effective market” are indispensable (37). Empirically, the government mainly facilitates the cultivation of market subjects through capital guidance, policy support, and standard guidance. It embodies the transition from the “ruling government” to the “management government” and then to the “service government”.

### 2.5. Actionable recommendations

Understanding the reform of the operation and management of sports venues is essential for the development of the sports industry. It is also the result of active exploration, joint practice, and positive interaction between the Chinese government and the market. This historical analysis identifies the changes and transformations through different periods and provides ideas and references for the development and reform of other industries and developing countries. History has proved that market-oriented reforms without restriction, supervision, protection, and cultivation are purely utopic, and government-led reform should respect the law of the market to play an effective role. As reform is affected by the changes in the relationship between the government and the market, one must carefully choose its application. It is estimated that the reform of the operation and management of sports venues will continue to adhere to the “market-oriented government-led” perspective, changing according to the needs of the time and reflecting the characteristics of China. It will help China to manifest its dream of becoming a leading sports nation.

### 2.6. Conclusions

The Chinese government has played an essential role in reforming the operation and management of sports venues. The “market-oriented government-led” model is a reform path of venue operation and management with unique characteristics about China and the current times, which is mainly manifested in three characteristics. First, the law of the market was respected and transformed from “administrative logic” to “market logic”. The second characteristic was to create an institutional environment to transition from “policy-driven development” to “systematic development”. The third characteristic was to cultivate the market entities and transition from “empowered enterprises” to “energized enterprises”. History has proven that market-oriented reforms lacking restrictions, supervision, protection, and cultivation are pure utopias, and the government's action can only play an effective role under the conditions of respecting the laws of the market.

## References

[1] Chinese-Sports-Almanac-Editorial-Committee. Chinese Sports almanac (1979). Beijing: People's Sports Press (1981).

[2] Chen Y. Study on supply of large-scale sports venues facilities. Wuhan: Central China Normal University Press (2011).

[3] Yuan W Li Z. Sports history of the people's republic of China (1949–1999). Beijing: China Book Publishing House (2002).

[4] Wan L. Resource utilization and operation management of stadiums and gymnasiums. Huazhong university of science and technology press: Wuhan (2010).

[5] Sun C Wang J Gao S. Study on the policy of sports facilities in China in 17 years after the establishment of PRC. J Shenyang Sport Univ. (2013) 32(6):70–3. 10.3969/j.issn.1004-0560.2013.06.018

[6] Yan C. Institutional reform and modern nation-building: since establishmen of PRC. Xuehai. (2019) 30(2):42–51. 10.16091/j.cnki.cn32-1308/c.2019.02.006

[7] CCCPC. Communique of the Third Session of the Eleventh Central Committee of the Communist Party of China1978 23 May, 2021. Available at: http://cpc.people.com.cn/GB/64162/64168/64563/65371/4441902.html

[8] CCCPC. Notice of the Central Committee of the Communist Party of China on Further Development of Sports1984 30 June, 2021. Available at: http://www.olympic.cn/rule_code/code/2004/0426/26065.html

[9] Chen Y Wang J Liu C. A historical review of China's sports venues supplies since establishment of PRC. J Xi'an Physical Edu Univ. (2013) 30(4):411–8. 10.16063/j.cnki.issn1001-747x.2013.04.017

[10] Liu D Bai J. Deng Xiaoping theory and China's sports reform twenty years of China's sports reform. Beijing: People's Sports Press (2001).

[11] Zhang W Zhang W. Discussion on several issues of economic responsibility evaluation of sports venues system. China Sport Sci. (1992) 12(3):19–22.

[12] Zhao G Lei L. Introduction to stadium operation and management. Beijing: Beijing Sport University Press (2007).

[13] Li L. Research on the financial guarantee of China's sports public undertakings. Wuhan: Wuhan University Press (2015).

[14] CCCPC, State-Council-of-PRC. Guiding Opinions on Promoting the Reform of Public Institutions by Classification 2011 6 Jaurary, 2022. Available at: http://www.gov.cn/gongbao/content/2012/content_2121699.htm

[15] Gong X Jin B Jia S. Shanghai Almanac 1998. Shanghai: Shanghai Almanac Press (1998).

[16] Wang Z Liang J Yang X. Investment and financing mode innovation and post-competition operation of Beijing new Olympic sports venues. China Sport Sci. (2012) 32(3):3–9. 10.16469/j.css.2012.03.004

[17] PCSC. Opinions on Cultivating the Sports Market and Accelerating the Process of Sports Industrialization1993 30 June, 2021. Available at: https://www.pkulaw.com/chl/ff0cbedb310abaedbdfb.html

[18] PCSC. China Sports almanac 1994–1995. Beijing: China Sports Almanac Press (1996).

[19] PCSC. Opinions on Deepening the Reform of Sports1993 30 June, 2021. Available at: https://www.pkulaw.com/chl/9e029dcdd95b7ed7bdfb.html

[20] Wang D Liang Z Xu C Zhao L. The two benefits go together, don't eat the imperial grain and pay the public grain: The deepening reform of Shanghai hongkou stadium. Beijing: The Situation of Sports Work (1994). 15–6.

[21] Chen W. Research on the system reform model of large-scale public stadiums in China. J Xi'an Physical Edu Univ. (2016) 33(3):295–8. 10.16063/j.cnki.issn1001-747x.2016.03.007

[22] Chen Y. Research on the diversified operation of large stadiums. J Sports Adult Educ. (2013) 29(4):1–7. 10.16419/j.cnki.42-1684/g8.2013.04.011

[23] Zhao D. Full development of wutaishan sports centre. Beijing: The Situation of Sports Work (1999). 9 p.

[24] State-Council-of-PRC. Public Cultural and Sports Facilities Regulations2003 23 May, 2021. Available at: http://www.gov.cn/zhengce/2020-12/26/content_5574621.htm

[25] Zhang L Huang H Wang Y. Review of the development of China's sports industry in 30 years of reform and opening-up. J Shanghai Univ Sport. (2008) 32(4):1–5. 10.16099/j.cnki.jsus.2008.04.002

[26] NPC. Outline of China's Twelfth Five-Year Plan for National Economic and Social Development2011 24 May, 2021. Available at: https://www.pkulaw.com/chl/603c7a7f2158b52bbdfb.html

[27] Chen Y Wang J. Study on the enterprise reform of operation and management of large-scale sports venues and gymnasium. China Sport Sci. (2015) 35(10):17–24. 10.16469/j.css.201510003

[28] GASC, NDRC, MPS, MF, MLR, MOHURD, et al. Opinions on Strengthening the Reform and Innovation of the Operation and Management of Large-scale Sports Venues and Improving the Level of Public Services2013 23 May, 2021. Available at: http://www.sport.org.cn/search/system/gfxwj/tyjj/2018/1115/193609.html

[29] State-Council-of-PRC. Several Opinions on Accelerating the Development of the Sports Industry and Promoting Sports Consumption2014 30 June, 2021. Available at: http://www.gov.cn/zhengce/content/2014-10/20/content_9152.htm

[30] GASC, NDRC. Action Plan for Further Promoting Sports Consumption (2019–2020)2019 30 June, 2021. Available at: http://www.gov.cn/zhengce/zhengceku/2019-11/15/content_5452392.htm

[31] Chen Y Chen L Li J Li J He Y Shi X. The effect of stadiums on urban renewal: american strategy and local enlightenment. J Shanghai Univ Sport. (2021) 45(2):78–89. 10.16099/j.sus.2021.02.009

[32] Chen Y Fang X. AComparative study on the level of public service provided by different operators of public sports venues. China Sport Sci. (2022) 42(08):85–97. 10.16469/j.css.202208008

[33] CCCPC. Create a New Situation in the Construction of Socialist Modernization1982 30 June, 2021. Available at: http://cpc.people.com.cn/GB/64162/64168/64565/65448/4526430.html

[34] CCCPC. Communique of the Third Plenary Session of the 14th Central Committee of the Communist Party of China1993 30 June, 2021. Available at: http://cpc.people.com.cn/GB/64162/64168/64567/65395/4441750.html

[35] CCCPC. Decision of the Central Committee of the Communist Party of China on Several Major Issues of Comprehensively Deepening Reform2013 30 June, 2021. Available at: http://cpc.people.com.cn/n/2013/1115/c64094-23559163.html

[36] Bao M. Set up a new mechanism combining the whole country system with market mechanism. China Sport Sci. (2018) 39(10):3–11. 10.16469/j.css.201810001

[37] Lin Y. China's experience: effective market and effective government are indispensable in economic development and transformation. Administrative Reform. (2017) 9(10):12–4. 10.14150/j.cnki.1674-7453.2017.10.002

